# Impact of H3K27 trimethylation loss in meningiomas: a meta-analysis

**DOI:** 10.1186/s40478-023-01615-9

**Published:** 2023-07-25

**Authors:** Gregory Cello, Ruchit V. Patel, James Tanner McMahon, Sandro Santagata, Wenya Linda Bi

**Affiliations:** 1 Department of Neurosurgery, Brigham and Women’s Hospital, Boston, MA USA; 2 Harvard Medical School, Boston, MA USA; 3 Department of Pathology, Brigham and Women’s Hospital, Boston, MA USA

**Keywords:** Meningioma, Epigenetic modification, H3K27me3, Prognosis, Genomics

## Abstract

Trimethylation of lysine 27 on histone 3 (H3K27me3) loss has been implicated in worse prognoses for patients with meningiomas. However, there have been challenges in measuring H3K27me3 loss, quantifying its impact, and interpreting its clinical utility. We conducted a systematic review across Pubmed, Embase, and Web of Science to identify studies examining H3K27me3 loss in meningioma. Clinical, histopathological, and immunohistochemistry (IHC) characteristics were aggregated. A meta-analysis was performed using a random-effects model to assess prevalence of H3K27me3 loss and meningioma recurrence risk. Study bias was characterized using the NIH Quality Assessment Tool and funnel plots. Nine publications met inclusion criteria with a total of 2376 meningioma cases. The prevalence of H3K27me3 loss was 16% (95% CI 0.09–0.27), with higher grade tumors associated with a significantly greater proportion of loss. H3K27me3 loss was more common in patients who were male, had recurrent meningiomas, or required adjuvant radiation therapy. Patients were 1.70 times more likely to have tumor recurrence with H3K27me3 loss (95% CI 1.35–2.15). The prevalence of H3K27me3 loss in WHO grade 2 and 3 meningiomas was found to be significantly greater in tissue samples less than five years old versus tissue of all ages and when a broader definition of IHC staining loss was applied. This analysis demonstrates that H3K27me3 loss significantly associates with more aggressive meningiomas. While differences in IHC and tumor tissue age have led to heterogeneity in studying H3K27me3 loss, a robust prognostic signal is present. Our findings suggest an opportunity to improve study design and standardize tissue processing to optimize clinical viability of this epigenetic marker.

**Supplementary Information:**

The online version contains supplementary material available at 10.1186/s40478-023-01615-9.

## Introduction

Meningiomas are the most common adult primary brain and spinal tumors in adults with a range of histological, molecular, and anatomical characteristics [1, 8, 16, 26]. The management of meningiomas is complicated by heterogenous patterns of recurrence and response to treatments such as adjuvant radiotherapy, limiting the ability to prognosticate clinical outcomes with precision in some tumors [3, 25, 38]. These factors are currently correlated with the World Health Organization (WHO) meningioma grade, which primarily relies on histopathological assessment [18]. However, the biological and clinical behavior of meningiomas can be variable and at times do not reflect their WHO grade [2, 28, 35]. Therefore, there has been a push to elucidate molecular markers and develop new classification schemes that more accurately reflect patient outcomes [2, 9, 21, 30].

One such genomic alteration that has recently been recognized as an indication of potentially worse prognosis is loss of lysine 27 trimethylation on histone 3 (H3K27me3) [18]. Histone modifications that regulate DNA expression have been implicated in the pathogenesis and progression of meningiomas, with initial studies connecting loss of H3K27me3 to poor patient outcomes and higher likelihood of recurrence [17]. This signal has been demonstrated in other nervous system tumors as well, with H3K27me3 loss occurring in an aggressive subset of malignant nerve sheath tumors, high-grade gliomas, and posterior fossa ependymomas [27, 29, 37]. Though evidence continues to grow on the mechanism underlying H3K27me3 loss, there has been significant variability in the reported incidence and clinical effect of this alteration in meningiomas. Additionally, methodological differences in how H3K27me3 loss is defined and quantified through immunohistochemistry (IHC) labeling could influence the validity of this marker for prognostication. Through this meta-analysis, we sought to assess the clinical relevance of H3K27me3 loss in meningioma as well as the impact of IHC and immunolabeling protocols utilized.

## Methods and materials

### Search strategy and study selection

We searched three databases, PubMed, Web of Science, and Embase, for all studies on H3K27me3 loss in meningiomas up to July 21, 2022. Additional publications were identified and included while data was being analyzed until March 26, 2023. A full list of search terms can be found in Additional file 1: Supplement 1. Preferred Reporting Items for Systematic Reviews and Meta-Analyses (PRISMA) guidelines were followed [33]. Inclusion criteria required immunohistochemical (IHC) staining of H3K27me3 in meningioma and at least five assessed cases. Studies were excluded if there was non-specific IHC staining for H3K27me3. Two reviewers (GC, RVP) independently screened abstracts and full texts through two rounds, with conflicts resolved by a third party (WLB).

### Data extraction and quality assessment

Publications were evaluated for clinical and histopathological variables of included meningioma cases, incidence of H3K27me3 loss, institutional IHC protocols for H3K27me3 staining, and meningioma recurrence risk after H3K27me3 loss. IHC protocols were broken down further to include tissue age, antibody dilution, slide incubation time, and definition of H3K27me3 loss. Multivariate adjusted hazard ratios were collected for tumor recurrence risk following H3K27me3 loss. Hazard ratios reported for univariate analyses only were excluded from the meta-analysis. The National Institutes of Health Quality Assessment Tool for observational cohort and cross-sectional studies (NIH-QAT) was used to assess the quality and risk of bias of included studies [19]. NIH-QAT is a 14-category inventory which evaluates domains from patient selection to reporting of outcome measures. Two reviewers (RVP, JTM) independently applied the NIH-QAT for each study and consensus was determined.

### Statistical analysis

Data aggregation and statistics were performed in R (R Foundation for Statistical Computing 4.2.1). All continuous variables are reported as mean ± standard deviation. Pearson’s chi-squared or Fisher’s exact test were utilized to compare categorical variables related to H3K27me3 loss. Quantitative parameters between H3K27me3 sub-groups were compared with the Mann–Whitney test. All statistical tests were two-tailed and *p* < 0.05 was considered significant.

For the meta-analysis, the prevalence of meningioma cases with H3K27me3 loss was determined for each study. Multivariate adjusted hazard ratios and 95% confidence intervals for tumor recurrence risk following H3K27me3 loss were collected from included studies. Data was pooled and effect size determined using a random-effects model with the weight of each study determined using an inverse variance method. Heterogeneity in the estimated prevalence of H3K27me3 loss and recurrence risk reported across studies was assessed using the *I*^2^ statistic, with *I*^2^ > 75% defined as the cutoff for substantial heterogeneity [12]. Publication bias was further assessed through funnel plots derived from the proportion of meningiomas with H3K27me3 loss and subsequent recurrence risk [10].

## Results

**Fig. 1:**
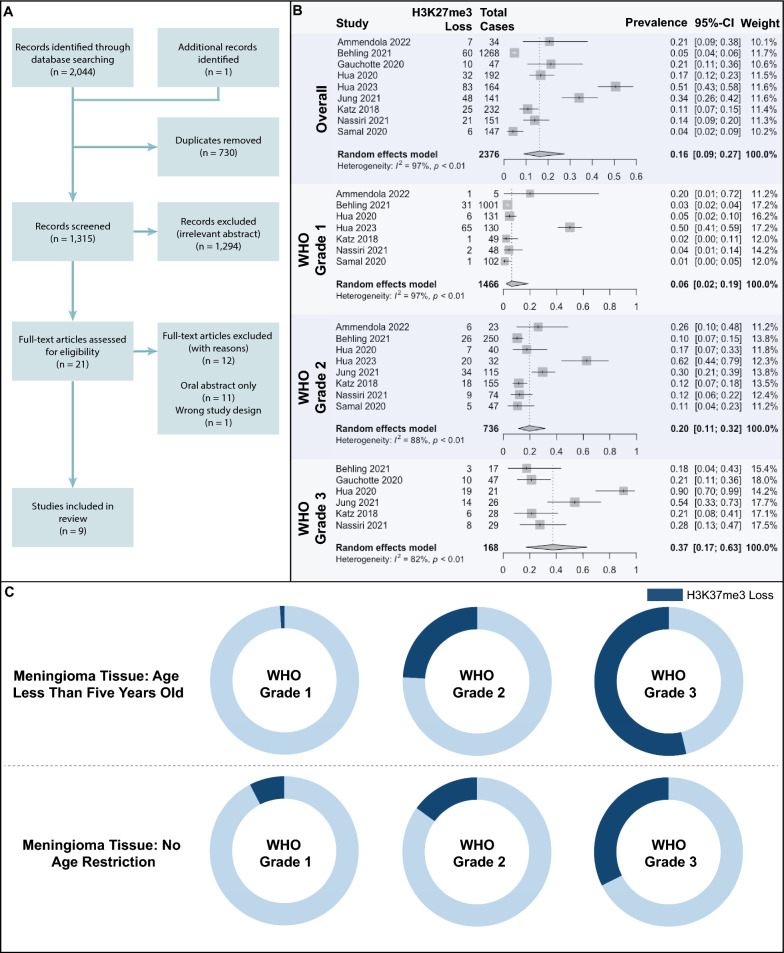
**A** PRISMA flowchart for study selection. **B** Meta-analysis demonstrating prevalence of H3K27me3 loss across included cases. Prevalence proportion was stratified by WHO grade. Pooled estimates were determined using a random-effects model and compared using a pairwise proportion z-score test. **C** H3K27me3 loss in meningiomas across WHO grades

We identified 2,045 unique articles with nine studies meeting inclusion criteria (Fig. 1A). A total of 2,811 patients with meningioma were identified, with 2,376 patients (809 male, 1,567 female) included for assessment of H3K27me3 loss (Table 1) [4, 7, 11, 13–15, 17, 24, 32]. Patients were excluded within these studies if there was inconclusive/technically unsatisfactory staining of meningioma pathology slides [4, 7, 11, 15, 24, 32] or if the paraffin block tissue was greater than five years old [15].

**Table 1:** Summary of meningioma cases included across all papers

	Meningioma Cases (n,%)				
	Original Cohort	Included Cohort	H3K27me3 Retained	H3K27me3 Ambiguous	H3K27me3 Lost
Ammendola et al.	39	34 (87.2)*	27 (69.2)	0 (-)	7 (18.0)
Behling et al.	1347	1268 (94.1)*	1208 (95.2)	0 (-)	60 (4.7)
Gauchotte et al.	66	47 (71.2)*	37 (78.7)	0 (-)	10 (21.3)
Hua et al. [14]	192	192 (100)	160 (83.3)	0 (-)	32 (16.7)
Hua et al. [13]	164	164 (100)	81 (49.4)	0 (-)	83 (50.6)
Jung et al.	468	141 (30.1)*^†^	93 (66.0)	0 (-)	48 (34.0)
Katz et al.	232	232 (100)	194 (83.6)	13 (5.6)^‡^	25 (10.8)
Nassiri et al.	181	151 (83.4)*	119 (78.8)	11 (7.3)^‡^	21 (13.9)
Samal et al.	149	147 (98.7)*	128 (87.1)	13 (8.8)^a^	6 (4.1)
Total	2811	2376 (84.5)	2047 (86.1)	37 (1.6)	292 (12.3)

*Reason for exclusion: inconclusive or technically unsatisfactory staining, ^†^Reason for exclusion: age of meningioma tissue, ^‡^Classified as H3K27me3 retained for analysis, ^a^Cases were unused in analysis

Overall, 292 patients with meningioma had loss of H3K27me3 expression. In an additional 37 cases, loss of H3K27me3 was ambiguous: 24 of those cases were defined as H3K27me3 retained and the remaining 13 were excluded from subsequent analysis (Table 1) based on IHC staining criteria.

Clinical and histopathological variables were extracted from studies that reported sub-stratification of meningioma cases by H3K27me3 loss (Table 2). A majority of cases assessed were newly diagnosed primary meningiomas (75.2%). Data on extent of resection was available for 28.0% of included cases, with 75.5% defined as gross total resection and 24.5% defined as subtotal resection. Simpson grade was available for 73.4% of cases: Simpson grade I, II, III, was achieved in 71.3% of cases and Simpson grade IV and V was achieved in 28.7% of cases. Adjuvant radiotherapy was subsequently administered to 14.8% of included patients.

**Table 2:** Distribution of meningioma H3K27me3 loss according to clinical and histopathological characteristics. Total counts only include cases if stratification was reported by publications

Parameter	Total n	H3K27me3 Retained, n (%)	H3K27me3 Lost, n (%)	*p*
*Sex*				
Male	695	578 (83.2)	117 (16.8)	< *0.001*
Female	1362	1235 (90.7)	127 (9.3)	
*Tumor*				
Primary	1365	1271 (93.1)	94 (6.9)	< *0.001*
Recurrent	451	316 (70.1)	135 (29.9)	
*Adjuvant radiotherapy*				
No	1279	1128 (88.2)	151 (11.8)	< *0.001*
Yes	222	162 (73.0)	60 (27.0)	
*Extent of resection*				
GTR	503	365 (72.6)	138 (27.4)	*0.79*
STR	163	120 (73.6)	43 (26.4)	
*Simpson grade*				
I/II/III	1243	1091 (87.8)	152 (12.2)	*0.40*
IV/V	500	446 (89.2)	54 (10.8)	
*WHO grade*				
I	1468	1363 (92.8)	105 (7.2)	< *0.001*
II	736	611 (83.0)	125 (17.0)	
III	168	108 (64.3)	60 (35.7)	
*Subtype*				
I				
Meningothelial	694	674 (97.1)	20 (2.9)	< *0.001*
Fibroblastic	111	105 (94.6)	6 (5.4)	
Transitional	154	150 (97.4)	4 (2.6)	
Psammomatous	59	57 (96.6)	2 (3.4)	
Angiomatous	33	33 (100)	0 (-)	
Microcystic	32	32 (100)	0 (-)	
Secretory	39	39 (100)	0 (-)	
Lymphocyte rich	2	2 (10)	0 (-)	
Metaplastic	17	17 (100%)	0 (-)	
II				
Atypical	411	372 (90.5)	39 (9.5)	
Chordoid	39	34 (87.2)	5 (12.8)	
Clear cell	2	2 (100)	0 (-)	
III				
Anaplastic	84	66 (78.6)	18 (21.4)	
Rhabdoid	6	5 (83.3)	1 (16.7)	
Papillary	1	1 (100)	0 (-)	
*Tissue age (< 5 years)*				
Yes	275	221 (80.4)	54 (19.6)	< *0.001*
No	2088	1850 (88.6)	238 (11.4)	
*Staining loss threshold*				
> 50% Absent	380	290 (76.3)	90 (23.7)	< *0.001*
> 95% Absent	1983	1781 (89.8)	202 (10.2)	

Chi-square test of proportions. GTR: Gross-total resection, STR: Sub-total resection, WHO: World Health Organization

A majority of articles assigned meningioma grade using the 2016 WHO criteria, with one study using the 2021 WHO criteria. 61.9% of meningiomas were grade 1, 31.0% were grade 2, and 7.1% were grade 3 (Table 2). Three of nine included papers further stratified meningioma subtypes within each WHO grade (71.0% of included cases). The most common subtypes analyzed were meningothelial (694/1684 cases, 41.2%) and transitional (154/1684 cases, 9.1%).

### H3K27me3 immunohistochemistry and quantification

The nine included studies were conducted at unique institutions with variations in IHC methods (Table 3) [4, 7, 11, 13–15, 17, 24, 32]. H3K27me3 loss was evaluated on either whole slides or tissue microarrays (TMA) across studies. Whole slide sections ranged from 3 to 5 μm in thickness and TMA cores ranged from 2 to 5 mm in diameter. All studies used rabbit derived H3K27me3 staining antibodies: rabbit monoclonal H3K27me3 antibody C36B11 (Cell Signaling, Danvers, MA, USA) or rabbit polyclonal H3K27me3 antibody HPA003916 (Sigma-Aldrich, St. Louis, MO, USA). The staining process utilized ranged both in incubation time (one hour to overnight) and antibody dilutions utilized (1:100–1:700).

**Table 3:** H3K27me3 IHC staining protocols in included studies

	Study location	IHC evaluation technique	TMA sample size	Whole slide sample size	Tissue age exclusion	Oldest included tissue age (years)	Antibody dilution	Antibody incubation time	Definition of H3K27me3 loss
Ammendola et al. [4]	Italy	Whole Slides	–	4 μm sections	None	Not Reported	1:200	Not Reported	Staining absent in > 95% of tumor cells
Behling et al. [7]	Germany	TMA, Whole Slides	2 × 1 mm diameter cores	4 μm sections	None	17	1:200	Not Reported	Staining absent in all tumor cells
Gauchotte et al. [11]	France	Whole Slides	–	Not Reported	None	30	1:100	1 h	Staining absent in > 50% of tumor cells
Hua et al. [14]	China	Whole Slides	–	4 μm sections	None	Not Reported	1:100	2 h	Staining absent in > 50% of tumor cells
Hua et al. [13]	China	Whole Slides	–	Not Reported	None	13	1:700	Not Reported	Staining absent in all tumor cells
Jung et al. [15]	Republic of Korea	TMA, Whole slides	2 mm diameter cores	3 μm sections	> 5 years	5	1:100	2 h	Staining absent in > 55% of tumor cells
Katz et al. [17]	Germany, United States of America	TMA, Whole slides	2 × 2 mm diameter cores	4 μm sections	None	Not Reported	1:100	2 h	Staining absent in all tumor cells
Nassiri et al. [23]	Canada	Whole Slides	–	5 μm sections	None	21	1:200	Overnight	Staining absent in all tumor cells
Samal et al. [3]	India	TMA, Whole Slides	1–3 × 5 mm diameter cores	3–4 μm sections	None	5	1:500	2 h	Staining absent in all tumor cells

**Fig. 2:**
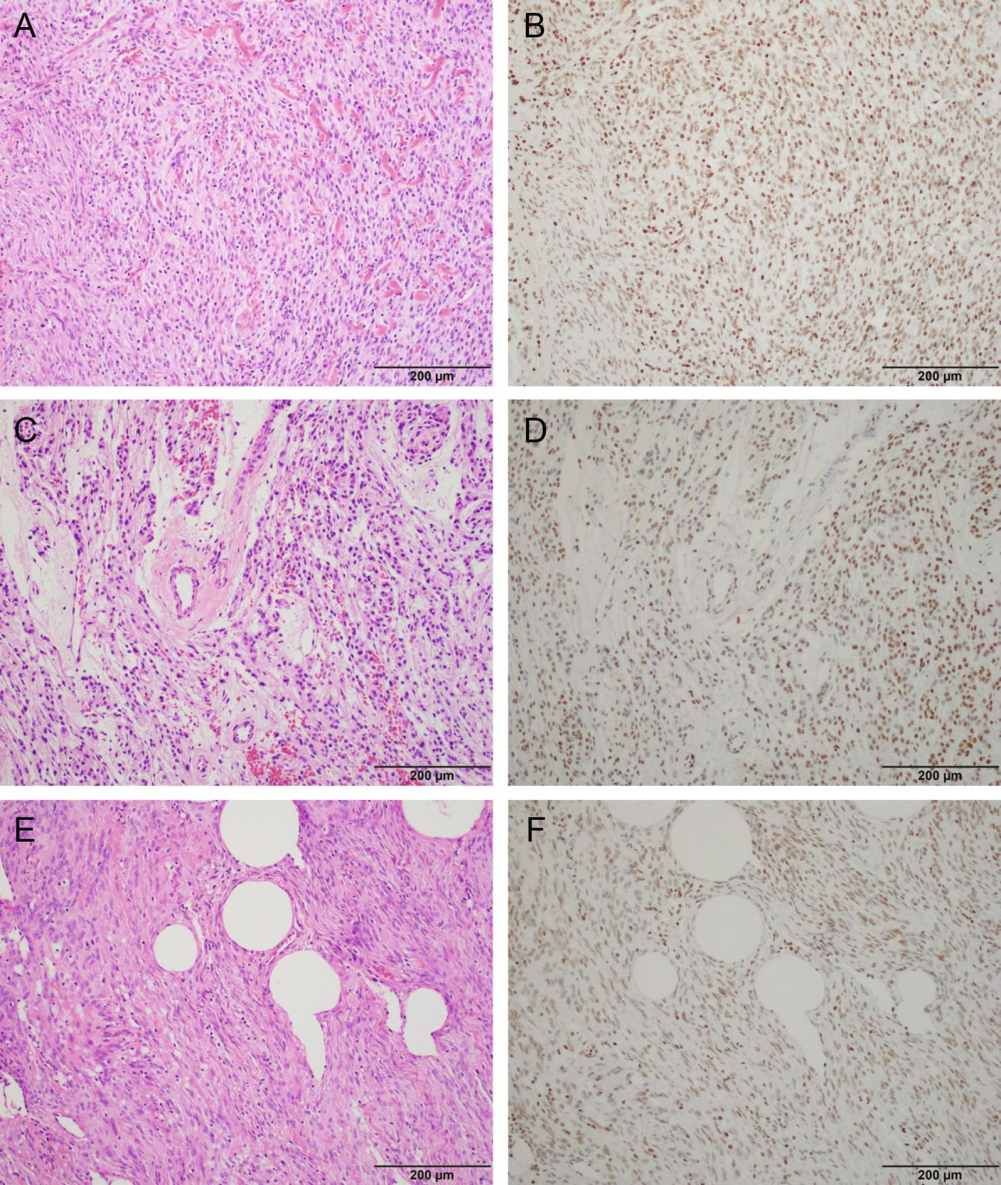
Representative hematoxylin and eosin staining (**A**, **C**, **E**) and paired H3K27me3 immunolabeling (**B**, **D**, **E**) in meningioma. Nuclear and endothelial staining is robust in B, indicating retention of H3K27me3 expression. **D** and **F** show decreased staining for H3K27me3 but heterogenous, still classified as retained

Different definitions were adopted for H3K27me3 loss. Six studies defined H3K27me3 loss as absent staining in 95–100% tumor cells in the field of view while the remaining three used absent staining in 50–55% of tumor cells. Representative images of these staining patterns collected from meningioma tissue at Brigham and Women’s Hospital can be seen in Fig. 2.

Finally, significant variability existed in the age of meningioma tissue evaluated across studies, ranging from 1–30 years old (Table 3). Two studies used meningioma tissue samples that were less than 5 years old [15, 32], with only one study pre-defining a strict tissue age cutoff of 5 years [15].

### Distribution of H3K27me3 loss

Across the nine included studies, the pooled prevalence of meningioma H3K27me3 loss was 16% (95% CI 9–27%). There was significant heterogeneity in this estimate (*I*^2^ = 97%), with point prevalence ranging from 4% [32] to 51% [13] (Fig. 1B).

As each study included a different proportion of meningiomas per WHO grade, H3K27me3 loss was examined within each tumor grade. The proportion of tumors with H3K27me3 loss significantly increased with higher WHO grade: 6% (95% CI 2–19%) of grade 1 tumors had H3K27me3 loss compared to 20% (95% CI 11–32%) of grade 2 and 37% (95% CI 17–63%) of grade 3 tumors (*p* < 0.001). Significant heterogeneity remained present between studies in the reported prevalence of H3K27me3 loss in WHO grade 1 (*I*^2^ = 97%), grade 2 (*I*^2^ = 88%), and grade 3 (*I*^2^ = 82%) tumors.

A significant modifier of reported H3K27me3 loss was the age of tissue samples included. Across all included cases, 19.6% of fresher tissue samples (age less than five years old) had H3K27me3 loss versus 11.4% of tissue samples without an age restriction (*p* < 0.001, Table 2). This trend was enhanced when sub-analyzing meningioma samples by WHO grade. Amongst WHO grade 2 meningiomas, 24.1% of tumor tissue less than 5 years old showed H3K27me3 loss versus 15.0% of non-age restricted tissue (*p* = 0.006). Similarly, for WHO grade 3 meningiomas, H3K27me3 loss was noted in 52.8% of tissue samples less than 5 years old compared to 32.4% across all age tissue (*p* = 0.036). Additionally, the definition of H3K27me3 loss based on IHC staining thresholds impacted the reported prevalence of H3K27me3 loss. 23.7% of cases across 3 studies were called with H3K27me3 loss when staining loss was observed in > 50% of tumor cells while 10.2% of cases across 6 studies were deemed to harbor H3K27me3 loss when a more stringent > 95% staining loss threshold was met (*p* < 0.001). Stringency of staining thresholds similarly impacted rates of reported H3K27me3 loss across WHO grade. For WHO grade 2 meningiomas, 26.5% of tumors had H3K27me3 loss with a > 50% loss staining threshold versus 14.5% with a > 95% loss staining threshold (*p* < 0.001). For WHO grade 3 meningiomas, 45.7% had H3K27me3 loss at a > 50% loss staining threshold compared to 23.0% at a > 95% threshold (*p* = 0.002). Analysis of the impact of tissue age and definition of H3K27me3 loss on the reported prevalence of WHO grade 1 meningiomas was limited due to sample size (Fig. 1C, Table 4). IHC parameters pertaining to antibody dilution did not significantly influence the proportion of cases with H3K27me3 loss.

**Table 4:** Distribution of meningioma H3K27me3 loss across different tissue specimen ages and staining loss thresholds after sub-dividing by WHO Grade

Parameter	Total, n	H3K27me3 Retained, n (%)	H3K27me3 Lost, n (%)	*p*
*WHO grade 1*				
Tissue age < 5 Years	102	101 (99.0)	1 (1.0)	0.012
Any tissue age	1366	1262 (92.4)	104 (7.6)	
*WHO grade 2*				
Tissue Age < 5 Years	162	123 (75.9)	39 (24.1)	0.006
Any tissue age	574	488 (85.0)	86 (15.0)	
*WHO grade 3*				
Tissue age < 5 Years	26	12 (46.2)	14 (53.8)	0.036
Any tissue age	142	96 (67.6)	46 (32.4)	
*WHO grade 1*				
Staining Absent > 50%	131	125 (95.4)	6 (4.6)	0.258
Staining Absent > 95%	1400	1299 (92.8)	101 (7.2)	
*WHO grade 2*				
Staining Absent > 50%	155	114 (73.5)	41 (26.5)	< 0.001
Staining Absent > 95%	581	497 (86.5)	84 (14.5)	
*WHO grade 3*				
Staining Absent > 50%	94	51 (54.3)	43 (45.7)	0.002
Staining Absent > 95%	74	57 (77.0)	17 (23.0)	

Chi-square test of proportions. WHO: World Health Organization

In addition to tissue age, definition of H3K27me3 loss, and WHO grade, male sex, recurrent tumor status, and receipt of adjuvant radiation were also associated with meningioma H3K27me3 loss in univariate analysis (Table 2). Male patients experienced a significantly higher loss of H3K27me3 at 16.8% compared to female patients at 9.3% (*p* < 0.001). H3K27me3 loss was significantly more common in recurrent meningiomas versus primary tumors (29.9% versus 6.9%, *p* < 0.001). Further, patients who subsequently received adjuvant radiotherapy after resection had greater H3K27me3 loss versus those did not receive adjuvant radiotherapy after resection, reflecting the enrichment of high-grade tumors in this population (*p* < 0.001). The extent of meningioma resection was not associated with H3K27me3 loss.

### Meningioma recurrence risk with H3K27me3 loss

**Fig. 3:**
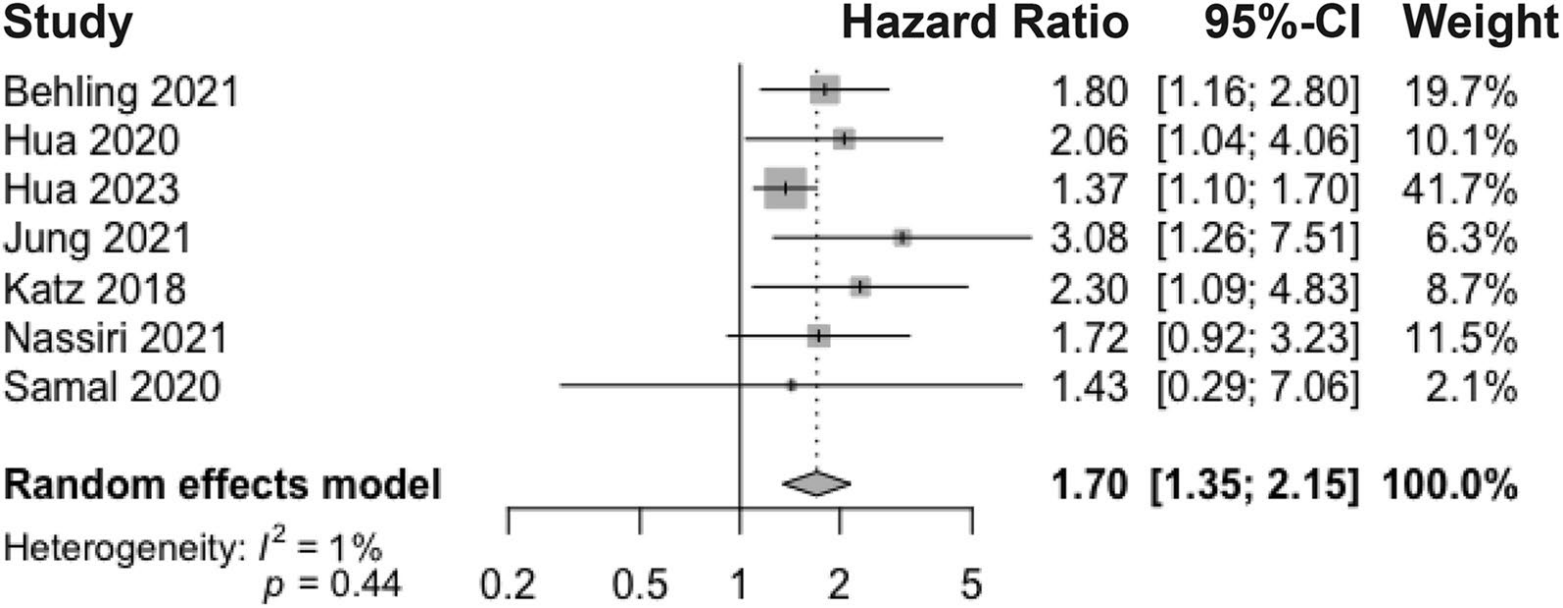
Meta-analysis of multivariate adjusted hazard ratios for meningioma recurrence following H3K27me3 loss. Pooled hazard ratio determined using a random-effects model

The pooled hazard ratio of meningioma recurrence with H3K27me3 loss was 1.70 (95% CI 1.35–2.15) across seven studies that reported multivariate hazard ratio data (Fig. 3). Low variability was observed across studies in the pooled recurrence hazard ratio (*I*^2^ = 1%). Variables that were controlled for included patient sex [7, 17, 24], age [7, 14, 15, 17, 24], tumor location [7], primary/recurrent tumor [7, 14, 15, 24], extent of resection/Simpson grade [7, 13, 17, 32], WHO grade [7, 13, 17, 24, 32], histopathological subtype [7], adjuvant radiotherapy administered [7], Ki-67/MIB1 proliferation index [7, 13–15], number of mitoses [15], and expression of the histone modification system EZH2 [32].

### Bias and quality evaluation

We applied the NIH-QAT tool as a metric of quality [19], with three studies rated as good and six as fair. Individual ratings across the 14 NIH-QAT domains are presented in Additional file 2: Supplementary Fig. 2A. All studies were performed as retrospective cohort studies where evaluators were not blinded. Studies had clearly defined study populations and recurrence measures. The greatest limitations in quality surrounded inclusion of sufficient follow-up time and either unclear or limited adjustment for confounding variables.

We evaluated bias in the pooled prevalence of H3K27me3 loss and meningioma recurrence risk using funnel plots and observed no significant skewing across included studies (Additional file 2: Supplementary Figs. 2B and 2C). Though there was slight asymmetry visually observed in the funnel plot for the prevalence of H3K27me3 loss, it was not statistically significant (*p* = 0.97). This reflects the variability in the reported proportion of H3K27me3 loss, even in studies with high precision (low standard errors) in their estimate [7, 15]. Differences in the relative proportion of high-grade tumors and tissue age of included samples likely contributed to the divergence in prevalence of H3K27me3 loss across included studies. The funnel plot for hazard ratios of meningioma recurrence after H3K27me3 loss also showed minimal asymmetry indicating limited bias in the estimate of recurrence risk across studies.

## Discussion

Genomic and molecular alterations have increasingly become important markers to better understand the presentation and clinical course of meningiomas. The loss of H3K27me3 has emerged as a major pathological subtype for certain central nervous system tumors such as diffuse midline gliomas and a feature predictive of prognosis [34]. As evidence grows elucidating the mechanism connecting H3K27me3 loss and oncogenesis, its role in the progression of meningiomas continues to be of interest. Through this meta-analysis, we demonstrate a signal connecting H3K27me3 loss to higher grade meningiomas and an increased risk of recurrence following tumor resection. Though a majority of included studies scored as fair quality on the NIH-QAT scale, we found variability in the reported estimates and the means of determining H3K27me3 loss, impacting the transability of this biomarker into clinical workflows.

The stratification of H3K27me3 loss across clinical variables revealed important trends connecting this marker to more aggressive tumors. While the pooled prevalence of H3K27me3 loss in meningioma was 16%, there were significant differences across WHO grades. WHO grade 3 meningiomas showed about six times the prevalence of H3K27me3 loss compared to WHO grade 1 meningiomas (37% versus 6%). Heterogeneity in the pooled prevalence of H3K27me3 loss was therefore likely driven by WHO grade differences in each study sample population, as the percent of WHO grade 3 meningiomas included ranged from 0 to 100% [4, 11, 32]. Other clinical variables which connotate aggressiveness also showed a significantly greater prevalence of H3K27me3 loss, including recurrent meningiomas and primary meningiomas with adjuvant radiotherapy. As a result, the relative proportion of recurrent meningiomas included in the study cohort likely also contributed to heterogeneity in the pooled prevalence of H3K27me3 loss. The significantly greater prevalence of H3K37me3 loss in males versus females is further consistent with the reported pattern of more aggressive meningiomas showing male predominance [6, 23, 31, 36].

The connection between more aggressive tumors and H3K27me3 loss was further evident in the pooled hazard ratio for meningioma recurrence. After multivariate adjustment, meningiomas with H3K27me3 loss had close to two times the risk of recurrence compared to those with retained H3K27me3. This is an effect size comparable to other previously described variables influencing meningioma recurrence risk, including incomplete surgical resection and presence of > 2 mitoses per 10 high-power fields [5]. While the overall heterogeneity was low between the seven studies reporting meningioma recurrence hazard ratios, there was a range in the variables each study adjusted for in their multivariate assessment. Only a subset of the seven studies controlled for variables with significant differences in H3K27me3 loss, including patient sex (3/7 studies), primary versus recurrent tumor (4/7 studies), and WHO grade (5/7 studies). Further, there was a wide range in the included patient sample size, from 141 to 1268 patients. The maximum follow-up period varied as well. Four studies had a similar follow-up period ranging from 15 to 17.5 years, but two included studies had a much shorter follow-up window between 3 and 6.5 years. Smaller studies coupled with uncontrolled variables and varying follow-up windows are all factors which can introduce heterogeneity into the estimate of recurrence risk. Additionally, many of these studies were not powered or designed to capture other clinical endpoints such as overall survival. This highlights the need for well-balanced studies across clinical characteristics with a clear plan for subgroup analysis and outcome measurement to assess the prognostic value of H3K27me3 loss more accurately.

A related barrier hampering the clinical utilization of H3K27me3 for meningioma is the reliable determination of methylation retention or loss. We considered multiple stages of the IHC and tissue processing pipeline to determine its impact on characterization of H3K27me3. In particular, the use of more fresh meningioma tissue (less than 5 years old) had a significant impact in the reported prevalence of H3K27me3 loss. This was seen across both WHO grade 2 and 3 tumors, with the prevalence of H3K27me3 loss almost doubling when comparing age restricted tissue with tissue of all ages. Additionally, with deterioration of antigenicity and tissue quality over time, older tissue fixed on slides has been shown to result in a higher incidence of poor or inconclusive staining [22]. Staining failure significantly reduced the number of included cases, with 435 meningioma samples removed from analysis due to unsatisfactory IHC. The heterogeneity in both staining failure as well as staining patterns within meningioma tissue represents a new challenge compared to the more unequivocal staining loss seen in other nervous system tumors such as diffuse midline gliomas [34]. Similar to tissue age, the application of a more stringent definition of staining loss (> 95% of tumor cells) reduced the reported prevalence of H3K27me3 loss. This highlights the need for additional institutional validation to determine if optimal prognostically significant IHC staining thresholds are present for defining H3K27me3 loss. Further, the antibody used for staining can have a dramatic impact on reported H3K27me3 loss. In the publication screening phase, we excluded one study which used an antibody that resulted in non-specific staining of inactive X chromosomes and peri-necrotic cells [20]. Standardization in tissue handling, staining methods, and quantification protocol will therefore be necessary steps to ensure H3K27me3 loss can be clinically useful.

Several limitations exist with this analysis, some of which are inherent to meta-analyses. The relatively few studies published assessing H3K27me3 loss in meningioma may impact the generalizability of the aggregated estimates. Further, pooled estimates are dependent on individual study reporting and outcomes. In some cases, such as when assessing recurrence risk following H3K27me3 loss, we were not able to collect a multivariate assessment from every study included. Differences in data handling and statistical adjustment can impact the accuracy of the pooled estimate. Finally, while we performed a univariate assessment of variables connected to H3K27me3 loss, we were unable to perform a multivariate regression due to missing data and the limited number of included studies. Nevertheless, we demonstrate a promising signal linking H3K27me3 loss to more aggressive meningiomas, an important step in characterizing the contribution of epigenetic modifications and developing prognostic markers that better reflect a patient’s clinical course.

## Supplementary Information

**Additional file 1.** List of database search terms.

**Additional file 2.** A. NIH-QAT evaluation of included studies. B. Funnel plot test for publication bias from pooled prevalence of H3K27me3 loss. C. Funnel plot test for publication bias from pooled multivariate adjusted hazard ratio of meningioma recurrence following H3K27me3 loss. A symmetric funnel plot forms when high precision studies fall close to the pooled meta-analysis estimate (top point of a funnel) while low precision studies have effect sizes that evenly distribute below or above the pooled estimate. Asymmetry in a funnel plot enables quantification of publication or reporting bias. Statistical testing of a funnel plot therefore indicates whether the estimated effect in the literature is biased or systematically skewed in a particular direction.

## Data Availability

All data presented in this study are available upon request from the corresponding author, WLB.
