# Supplementary material for: Impact of H3K27 trimethylation loss in meningiomas: a meta-analysis
Source: Acta Neuropathol Commun. 2023 Jul 25;11:122. doi: 10.1186/s40478-023-01615-9 (PMC10369842; doi:10.1186/s40478-023-01615-9)
Supplement: Supplementary file 1 — Additional file 1. List of database search terms. [file 40478_2023_1615_MOESM1_ESM.docx]

**Supplement 1: Database Search Terms**

(mening* OR meningioma) AND (H3K27me3 OR H3K27 trimethylation OR H3K27 methylation OR H3K27 OR H3 trimethylation OR H3 methylation OR H3 OR trimethylation OR methylation)
