# Supplementary material for: Impact of H3K27 trimethylation loss in meningiomas: a meta-analysis
Source: Acta Neuropathol Commun. 2023 Jul 25;11:122. doi: 10.1186/s40478-023-01615-9 (PMC10369842; doi:10.1186/s40478-023-01615-9)
Supplement: Supplementary file 2 — Additional file 2. A. NIH-QAT evaluation of included studies. B. Funnel plot test for publication bias from pooled prevalence of H3K27me3 loss. C. Funnel plot test for publication bias from pooled multivariate adjusted hazard ratio of meningioma recurrence following H3K27me3 loss. A symmetric funnel plot forms when high precision studies fall close to the pooled meta-analysis estimate (top point of a funnel) while low precision studies have effect sizes that evenly distribute below or above the pooled estimate. Asymmetry in a funnel plot enables quantification of publication or reporting bias. Statistical testing of a funnel plot therefore indicates whether the estimated effect in the literature is biased or systematically skewed in a particular direction. [file 40478_2023_1615_MOESM2_ESM.pdf]

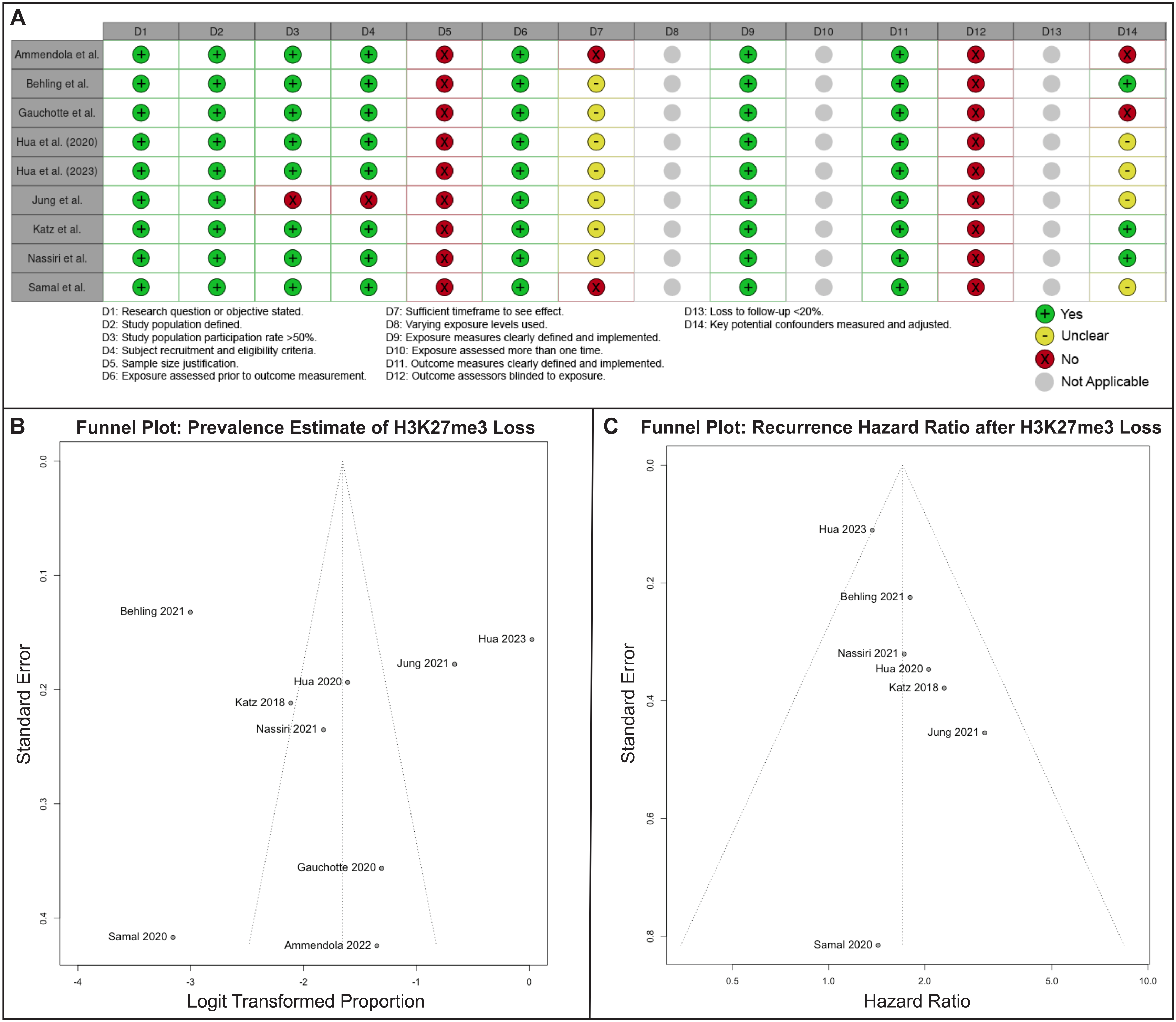
A
D1
D2
D3
D4
D5
D6
D7
D8
D9
D10
D11
D12
D13
D14
Ammendola et al.
Behling et al.
Gauchotte et al.
Hua et al. (2020)
Hua et al. (2023)
Jung et al.
Katz et al.
Nassiri et al.
Samal et al.
D1: Research question or objective stated.
D2: Study population defined.
D3: Study population participation rate >50%.
D4: Subject recruitment and eligibility criteria.
D5: Sample size justification.
D6: Exposure assessed prior to outcome measurement.
D7: Sufficient timeframe to see effect.
D8: Varying exposure levels used.
D9: Exposure measures clearly defined and implemented.
D10: Exposure assessed more than one time.
D11. Outcome measures clearly defined and implemented.
D12: Outcome assessors blinded to exposure.
D13: Loss to follow-up <20%.
D14: Key potential confounders measured and adjusted.
Yes
Unclear
No
Not Applicable
B
Funnel Plot: Prevalence Estimate of H3K27me3 Loss
Standard Error
Logit Transformed Proportion
Behling 2021
Hua 2023
Jung 2021
Hua 2020
Katz 2018
Nassiri 2021
Gauchotte 2020
Samal 2020
Ammendola 2022
C
Funnel Plot: Recurrence Hazard Ratio after H3K27me3 Loss
Standard Error
Hazard Ratio
Hua 2023
Behling 2021
Nassiri 2021
Hua 2020
Katz 2018
Jung 2021
Samal 2020
